# Effect of N Atom Substitution on Electronic Resonances: A 2D Photoelectron Spectroscopic and Computational Study of Anthracene, Acridine, and Phenazine Anions

**DOI:** 10.1021/acs.jpca.4c02756

**Published:** 2024-06-27

**Authors:** Stephen Slimak, Aude Lietard, Kenneth D. Jordan, Jan R. R. Verlet

**Affiliations:** † Department of Chemistry, University of Pittsburgh, Pittsburgh, Pennsylvania 15260, United States; ‡ Department of Chemistry, Durham University, Durham DH1 3LE, U.K.; § J. Heyrovský Institute of Physical Chemistry, Czech Academy of Sciences, Dolejškova 3, Prague 8 18223, Czech Republic

## Abstract

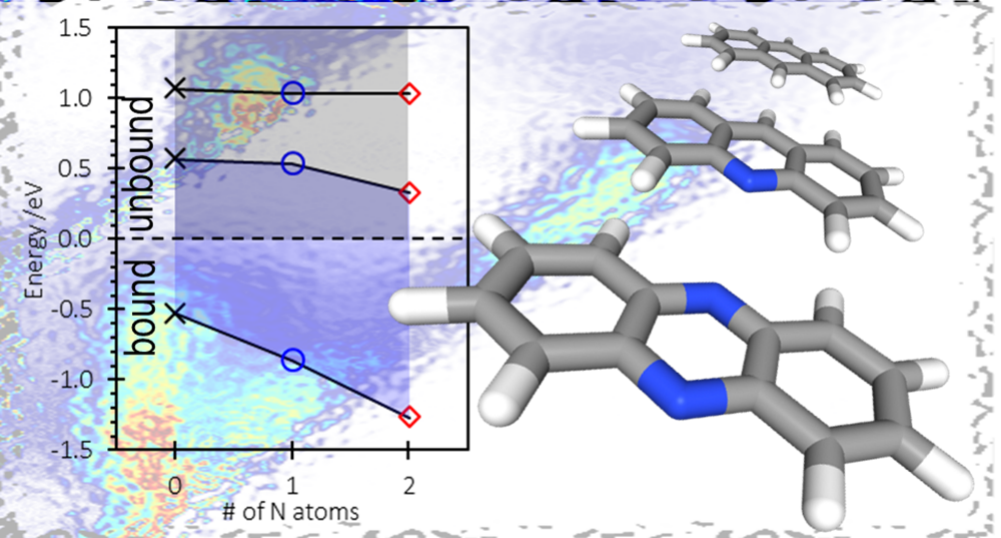

The accommodation of an excess electron by polycyclic aromatic hydrocarbons (PAHs) has important chemical and technological implications ranging from molecular electronics to charge balance in interstellar molecular clouds. Here, we use two-dimensional photoelectron spectroscopy and equation-of-motion coupled-cluster calculations of the radical anions of acridine (C_13_H_9_N^–^) and phenazine (C_12_H_8_N_2_^–^) and compare our results for these species to those for the anthracene anion (C_14_H_10_^–^). The calculations predict the observed resonances and additionally find low-energy two-particle-one-hole states, which are not immediately apparent in the spectra, and offer a slightly revised interpretation of the resonances in anthracene. The study of acridine and phenazine allows us to understand how N atom substitution affects electron accommodation. While the electron affinity associated with the ground state anion undergoes a sizable increase with the successive substitution of N atoms, the two lowest energy excited anion states are not affected significantly by the substitution. The net result is that there is an increase in the energy gap between the two lowest energy resonances and the bound ground electronic state of the radical anion from anthracene to acridine to phenazine. Based on an energy gap law for the rate of internal conversion, this increased gap makes ground state formation progressively less likely, as evidenced by the photoelectron spectra.

## Introduction

The accommodation of electrons in carbonous materials and specifically polycyclic aromatics (PAHs) is important in science and technology. For example, the flow of charge through conjugated π-systems underpins organic molecular electronic devices,^1−3^ and the charge balance in the molecular clouds in the interstellar medium is impacted directly by the carriers of negative charge being PAH anions rather than free electrons.^4−7^ The incorporation of heteroatoms within the PAH can lead to subtle changes in electronic properties that can influence the electron transport and accepting abilities of the PAH and more generally its chemistry. The most common substitutions are N and O atoms with the former being particularly common in the interstellar medium.^8−11^ Here, we focus on N-substituted PAHs, PANHs. Specifically, we consider the effect of N atom substitution on the electronic states of the radical anions of anthracene (C_14_H_10_) by probing the anions of acridine (C_13_H_9_N) and phenazine (C_12_H_8_N_2_). C_14_H_10_ is a small linear PAH with a positive electron affinity,^12−14^ and C_13_H_9_N and C_12_H_8_N_2_ offer mono- and di-N atom substitution on the central ring. Together, these closely related molecules provide an ideal case to assess and understand the changes that N atom substitution causes in prototypical PAH.

Fundamental to electron accommodation in PAHs or PANHs is the electronic states associated with their respective anions. In isolation, a collision between a free electron and a closed-shell PAH/PANH leads to the formation of temporary negative ions when the kinetic energy of the incoming electron matches the energy of a temporary anion resonance.^15,16^ The temporary anions are inherently metastable to electron autodetachment. The time scale of autodetachment depends on how strongly the resonance is coupled to the continuum. For a one-particle (1p or shape) resonance, autodetachment typically occurs on a 10^–14^–10^–13^ s time scale. For a two-particle one-hole (2p1h) resonance lying energetically below its 1p1h parent, autodetachment is slower because of the requirement for the additional electron deexcitation that accompanies the electron loss and generally occurs on a 10^–13^–10^–11^ s time scale. Regardless of the electronic configuration of the resonance, nuclear dynamics take place on similar time scales and can therefore compete with autodetachment. Hence, the population in the resonance can access different parts of its corresponding potential energy surface (and the autodetachment lifetime can vary with the geometry). As a consequence, the kinetic energy of the outgoing electron produced in the autodetachment process will vary and offer a measure of the resonance dynamics. Nuclear dynamics also opens the possibility of internal conversion through conical intersections,^17^ which will also be reflected in the kinetic energy of the outgoing electron. In the extreme case, internal conversion can lead to the formation of the ground electronic state of the radical anion which, for most PAHs (except for naphthalene), is electronically bound. Electron emission can still take place in the gas phase because the total energy imparted by the electron collision remains larger than the electron affinity of the PAH. The electron emission process in this case is statistical and typically takes place over 10^–10^–10^–3^ s (dependent on electron affinity and degrees of freedom of the PAH) and has a Boltzmann energy distribution.^18^

Electron resonances can be probed experimentally by electron–molecule scattering experiments such as electron transmission spectroscopy and electron excitation spectroscopy that probes the location of resonances, and electron energy loss spectroscopy that measures the outgoing electron kinetic energy.^16,19,20^ The latter can additionally be performed by scanning the incoming electron kinetic energy, thus offering 2D electron energy loss spectra (electron counts as a function of both incoming and outgoing electron kinetic energy).^21−24^ While such measurements are the most direct probe of electronic resonances, they have not yet been extended to N-substituted PAHs. As an alternative, we have recently developed 2D photoelectron spectroscopy from the anion as a complementary measure of electron resonances,^25,26^ including those of PAHs.^27−29^ In this approach, photons with energies in excess of the electron affinity of the neutral molecule, *h*ν > EA, interact with the ground state anion leading either to direct detachment into the molecule + e^–^ continuum or excitation of electron impact resonances. As the resonances are those accessed in electron-PAH/PANH collisions, the kinetic energy distribution of the outgoing photoelectron carries information similar to that obtained by electron energy loss spectroscopy. Hence, by scanning *h*ν across the continuum, data analogous to 2D electron energy loss spectroscopy can be obtained.^26^ The two important differences are that the initial geometries differ (anion vs neutral) and the excitation selection rules differ (photon vs electron excitation). However, for PAH/PANHs, the differences in the geometries of the ground states of the anionic and neutral molecules are relatively small, and the selection rules in the case of 2D electron energy loss measurements are dominated by the electric dipole interaction in the energy range considered here. Hence, the analogy holds well, with key added benefits of 2D photoelectron spectroscopy: when performed using photoelectron imaging,^30^ the experiment is sensitive to very low-energy electrons (unlike electron energy loss spectroscopy) and offers photoelectron angular distributions that are sensitive to the nature of the orbital from which the electron is emitted.^31,32^

From a theoretical perspective, C_14_H_10_ is interesting because of its well-known electronic structure and the fact that the energies of the 1p anion resonances can be fairly accurately predicted by using the pairing theorem.^28^ However, the pairing theorem does not hold for the N-substituted species, making electronic structure calculations of the energies especially valuable. Computation of electronic resonances requires the use of a method that deals with the fact that these states are embedded in the electron detachment continuum.^33^ In the present work, we make use of the equation-of-motion coupled-cluster method^34,35^ combined with the stabilization method^36,37^ to characterize the anion states. The details of the calculations are provided below.

The electronic resonances and excited anionic states of C_14_H_10_ have been studied over several decades. Absorption spectra of the radical anion, C_14_H_10_^–^, have been measured in solution^38^ and in cold molecular matrices.^39,40^ In the gas phase, the electronic resonances have been measured by electron transmission spectroscopy^41^ and electron excitation spectroscopy.^20^ There have also been several photoelectron spectroscopic studies on C_14_H_10_^–^ and its clusters,^12,13,42^ including at very high resolution^14^ and using 2D photoelectron spectroscopy.^28,43^ The resonances were also considered computationally. Below, we offer a slight revision to the assignment of the electronic resonances of C_14_H_10_. The photoelectron spectra of C_13_H_9_N^–^ and C_12_H_8_N_2_^–^ at specific photon energies have also been reported,^44−46^ but their 2D photoelectron spectra have not been analyzed and considered in the context of C_14_H_10_^–^; this comparison is the main motivation for the current work.

## Methods

### Experimental Details

The experiment has been described in detail elsewhere.^47^ Briefly, anions were generated by pulsing the vapor pressure of heated (∼200 °C) samples of anthracene, acridine, and phenazine (Sigma-Aldrich) using a pulsed valve (Even Lavie^48^) with Ar as backing gas into vacuum and crossing the expansion with 300 eV electrons from an electron gun. The resulting anions were extracted using a time-of-flight mass spectrometer,^49^ and at its focus, mass-selected ion packets were intersected with the output from a Nd:YAG pumped optical parametric oscillator (Continuum). The resulting photoelectrons were collected in a velocity-map imaging^30^ spectrometer which had a resolution of ∼3% of the electron’s kinetic energy.^47^ Images were reconstructed to offer photoelectron spectra and angular distributions using polar onion peeling.^50^ The photoelectron angular distributions, defined as the photoelectron signal emitted at the angle, θ, relative to the polarization vector of the light, have been quantified using the anisotropy parameter, β_2_, which scales from +2 (corresponding to a cos^2^ θ distribution) to −1 (corresponding to a sin^2^ θ distribution).^31^

### Computational Details

The energies of the anion states were calculated with the equation-of-motion coupled-cluster singles and doubles method (EOM-CCSD)^34,35^ using the cc-pVTZ(-f,-d) basis set augmented with diffuse p functions on the carbon and nitrogen atoms. The “-f, -d” indicates that the carbon and nitrogen f functions and the hydrogen d functions in the cc-pVTZ basis set^51^ were omitted. Because all the anion states other than the ground states are unbound, the EOM-CCSD calculations were coupled with the stabilization method^36,37^ in which the eigenvalues corresponding to the excess electron system are considered as a function of a scale parameter that scales the exponent of the diffuse p functions. In a diabatic picture, the excess electron states consist of discrete states that correspond to the anion states in the absence of coupling to the scattering continuum and discretized continuum (DC) levels in the unbound region. The energies of the discrete states are relatively insensitive to the scale factor, while the DC levels drop rapidly in energy as the scaled functions become more diffuse. As the scale factor is varied, the two types of levels undergo avoided crossings. We use generalized Padé approximants^52,53^ to analytically continue the energy vs scale parameter curves into the complex plane to determine the complex resonance energies. Here, we are primarily interested in the real parts of the energies that give the resonance positions.

The EOM-CCSD calculations were carried out at both the geometries of the neutral molecules and the geometries of their ground state anions. The geometries were obtained from optimizations using the B3LYP^54−56^ density functional method together with the cc-pVTZ basis set.^51^ Photoelectron spectra were simulated using a Franck–Condon simulation between the anion and neutral ground states, employing harmonic frequencies and wave functions calculated at the B3LYP/cc-pVTZ level of theory. The spectral lines have been convoluted using a Gaussian function with a full width at half-maximum of 16.7 meV (135 cm^–1^). Displacement vectors have been visualized using jmol.^57^

## Results

### 2D Photoelectron and β_2_ Spectra of Acridine and Phenazine Radical Anions

**Figure 1:**
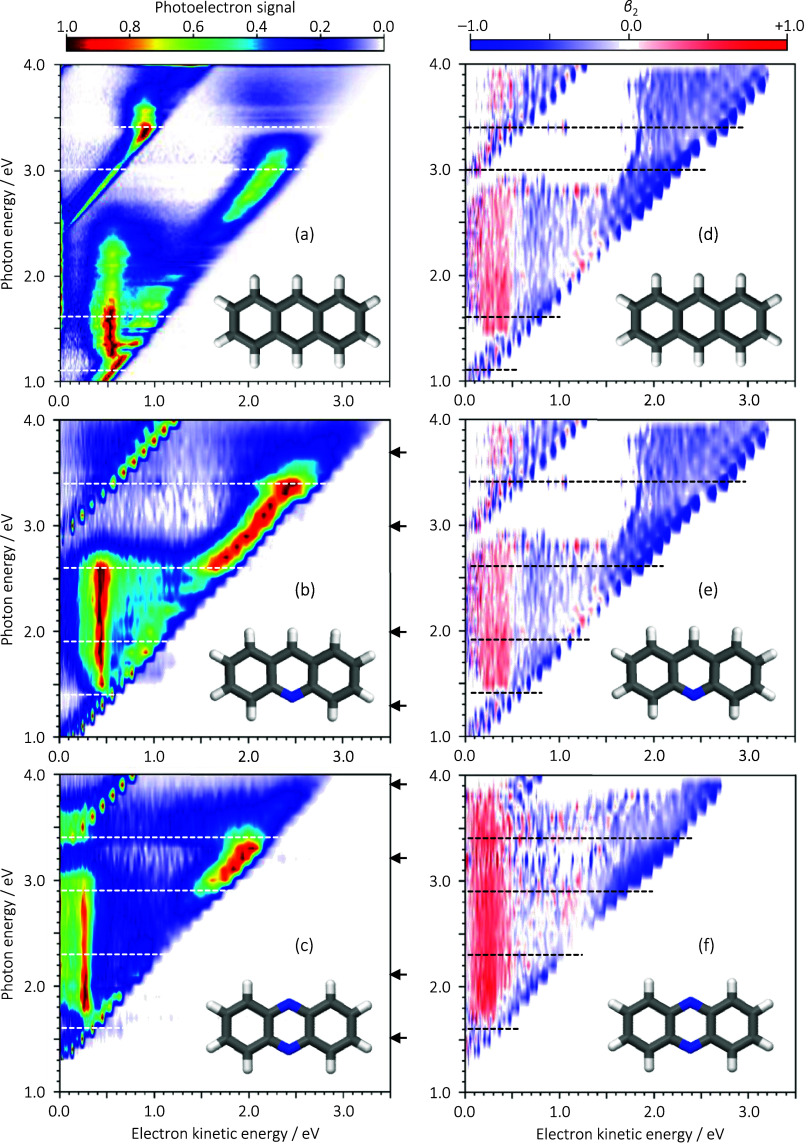
2D photoelectron spectra of (a) anthracene, (b) acridine, and (c) phenazine, with their respective structure insets and corresponding 2D β_2_ spectra in (d–f), respectively. Locations of resonances are indicated by dashed horizontal lines. Arrows on the right of the 2D photoelectron spectra indicate that the photon energies used in Figure 2. (a) has been adapted from ref (28), Copyright [2020], with the permission of AIP Publishing.

**Figure 2:**
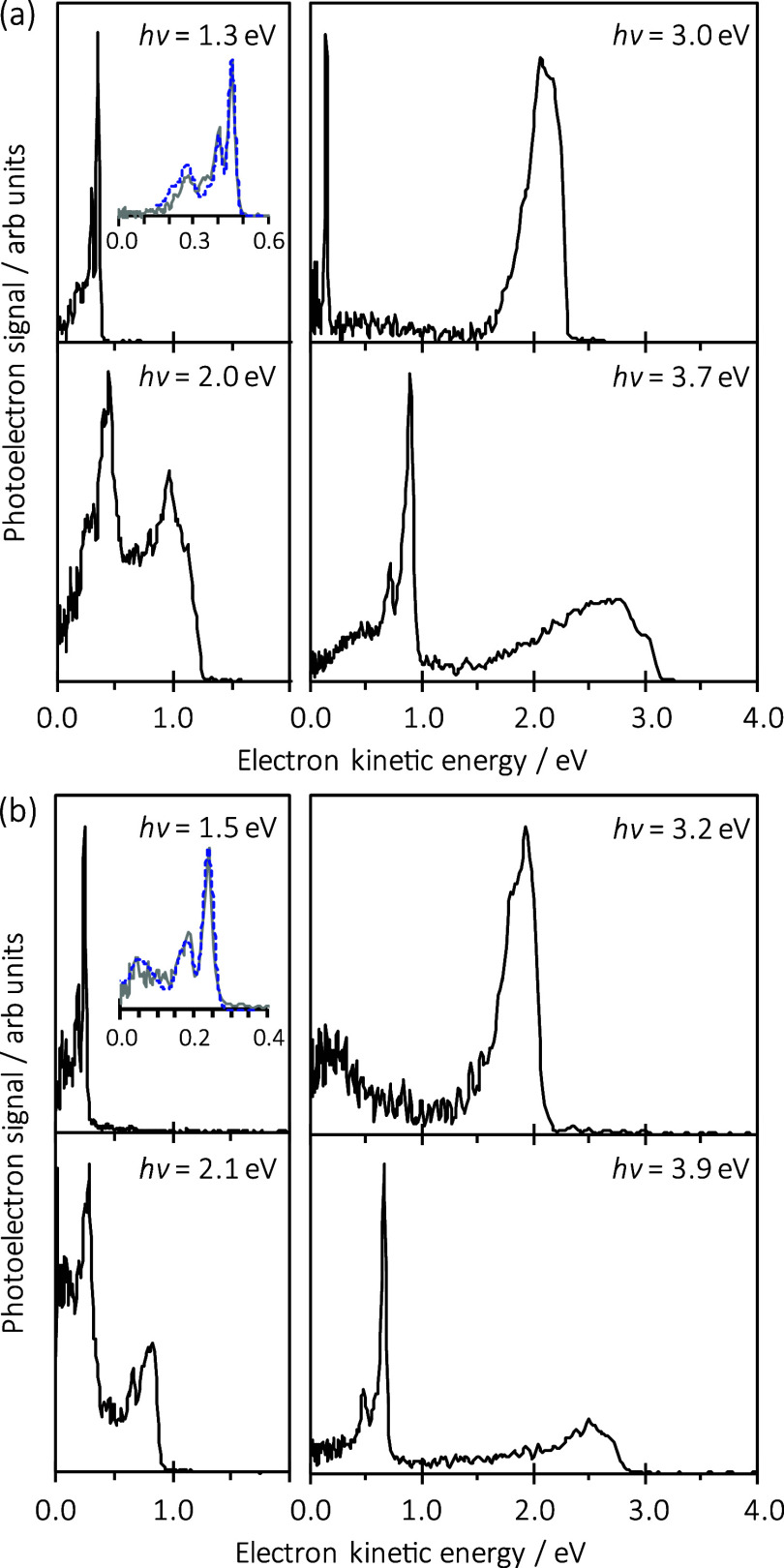
Photoelectron spectra of (a) acridine and (b) phenazine taken at representative photon energies, *h*ν, as indicated in Figure 1b,c, respectively. Insets highlight the low-energy region of the direct detachment (gray solid line), which shows vibrational progressions associated with the neutral ground state. The dashed blue lines in these insets are computed photoelectron spectra.

Figure 1a–c shows the 2D photoelectron spectra of anthracene, acridine, and phenazine anions, with molecular structures shown for each. The 2D spectra are shown in the range of 1.0 ≤ *h*ν ≤ 4.0 eV in increments of 0.1 eV for acridine and phenazine and 0.025 eV for anthracene. Each photoelectron spectrum has been normalized to its maximum value. The 2D photoelectron spectrum of the anthracene anion, shown in Figure 1a, has been discussed previously^28^ and is included here predominantly as a reference to investigate the effect of N atom substitution although our new calculations also offer some additional insights into the resonances that are present in this molecule. The corresponding 2D β_2_ spectra for anthracene, acridine, and phenazine anions are shown in Figure 1d–f and support the consideration of the results below. Figure 2a,b shows representative spectra (i.e., horizontal slices of the 2D spectrum) at locations indicated by the horizontal arrows in Figure 1b,c, respectively.

**Figure 3:**
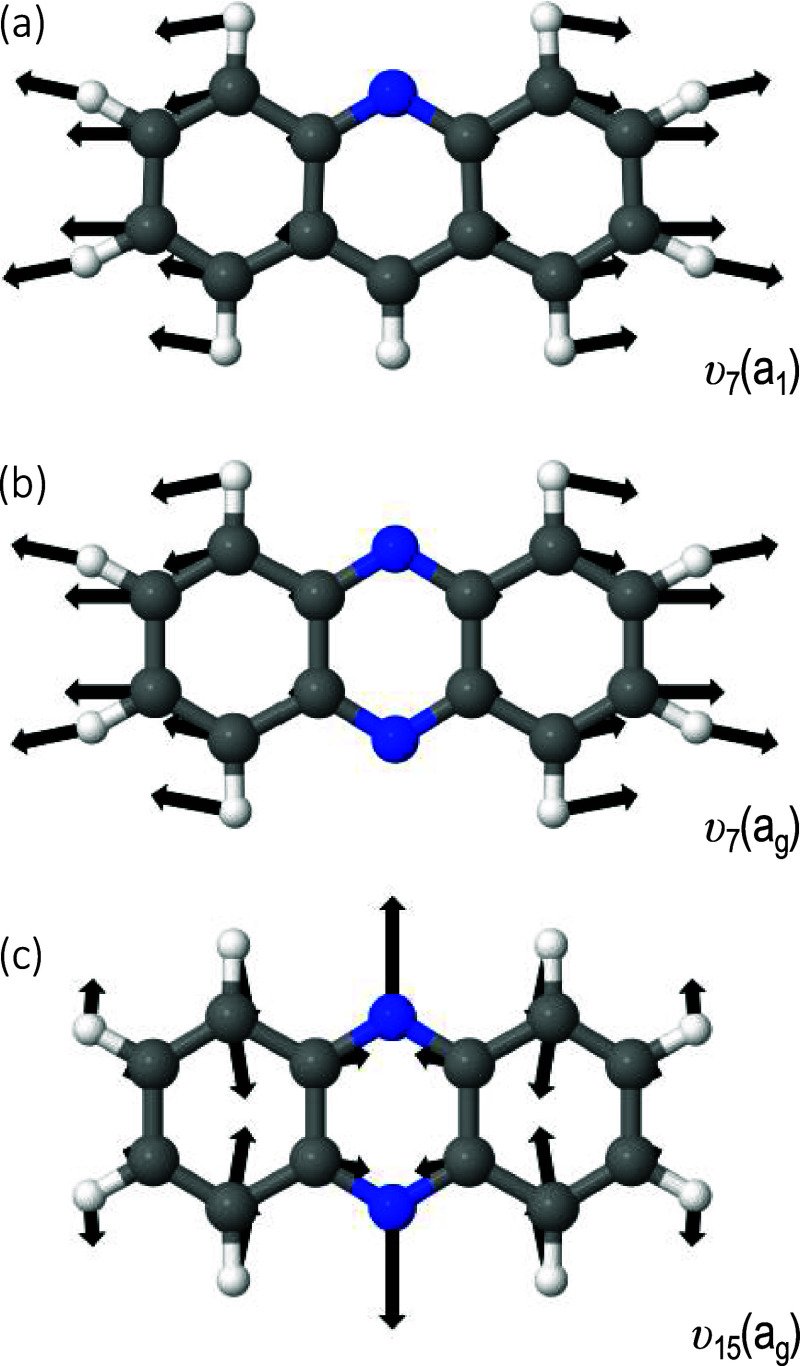
Displacement vectors of the dominant vibrational modes excited in the neutral following photodetachment: (a) υ_7_(a_1_) for acridine; (b) υ_7_(a_g_) and (c) υ_15_(a_g_) for phenazine.

The 2D photoelectron spectra show two main classes of features. Diagonal features with a unit gradient represent features whose eKE changes in line with the variation in *hv*. These arise from direct detachment of the anion ground state into the neutral + e^–^ continuum (i.e., photoelectric effect). As an example, for the acridine anion, two such features can be seen in Figure 1b: one with a *h*ν-intercept at 0.86 eV and the other with a *h*ν intercept of 2.86 eV. These correspond to anion photodetachment leaving the neutral in the ground electronic state and in the first excited (triplet) state of the neutral, respectively. The direct photoelectron spectra for both processes can be clearly seen in Figure 2a at *h*ν = 1.3 eV and *h*ν = 3.0 eV, respectively. These allow us to determine the adiabatic electron affinity of acridine to be 0.86 ± 0.01 eV and the S_0_–T_1_ gap to be 2.00 ± 0.02 eV, which is consistent with a previous measurement.^44^ The spectrum at *h*ν = 1.3 eV also allows us to identify a series of peaks with a spacing of 52 ± 2 meV (420 ± 20 cm^–1^), which corresponds to a vibrational progression in the neutral ground state arising from the Franck–Condon factors between the ground state of the anion and that of the neutral. The inset of the photoelectron spectrum at *h*ν = 1.3 eV also includes a calculated photoelectron spectrum, which shows excellent agreement and allows us to assign the dominant Franck–Condon active mode as the υ_7_(a_1_) mode with a computed frequency of 409 cm^–1^. The displacement vectors of the υ_7_(a_1_) mode are shown in Figure 3a.

The second class of features are those that do not follow the diagonal trend outlined above, and they typically appear in different ways in the 2D photoelectron spectrum. The first are signals that are at constant eKE with varying *h*ν. Such a feature is clearly visible for the acridine anion around eKE = 0.45 eV from *h*ν = 1.4 to 2.7 eV (see Figure 1b). The spectrum at *h*ν = 2.0 eV in Figure 2a is a representative example. The second way is through spectral changes in the shape of the photoelectron spectrum (i.e., changes in the Franck–Condon factors). Such changes can be seen in the photoelectron spectra with *h*ν ≥ 2.7 eV, with the spectrum at *h*ν = 3.0 eV in Figure 2a as a representative example for acridine. In this, the shape of the direct detachment appears broadened (beyond that expected from the poorer resolution at higher eKE). It again changes shape around *h*ν ≥ 3.4 eV as shown by the spectrum at *h*ν = 3.7 eV in Figure 2a.

The off-diagonal features come about from the excitation to anion resonances, which either have differing Franck–Condon factors with the final neutral states or can undergo dynamics, leading to the loss of energy in the outgoing electron. Hence, the appearance energies of these changes give some indication of the location of the anion resonances. This is further supported by the 2D β_2_ spectrum, which shows changes in the angular distributions that are consistent with changes observed in the 2D photoelectron spectra. In the case of acridine, Figure 1e, for example, while direct detachment is expected to be characterized by β_2_ < 0 (from the detachment from a π-orbital),^32^ the peak at constant eKE = 0.45 has β_2_ > 0. Additionally, in the “direct detachment” diagonal feature, there are sudden changes in either the sign or magnitude of β_2_. Such changes are exemplified in acridine at *h*ν = 2.0 eV (where β_2_ becomes much less negative and even slightly positive) and at *h*ν = 2.6 eV (where β_2_ suddenly becomes much more negative). As the photoelectron angular distribution is determined by the orbital from which the electron is lost and by the mechanism (be it photodetachment or autodetachment), these changes offer an additional indication of the presence and location of electronic resonances.^26,58−60^ The horizontal dashed lines in Figure 1b and its corresponding 2D β_2_ spectrum in Figure 1e highlight the location of resonances in acridine based on our observations of changes identified in either the 2D photoelectron or β_2_ spectra. The location of resonances have been determined by the onset at which clear changes in the photoelectron spectra can be noted as described in detail for anthracene in our previous study.^28^ The energetic onset of these transitions can generally be determined to within ±0.1 eV.

Figure 1c,f shows equivalent data for photodetachment from phenazine anions, and Figure 2b shows representative photoelectron spectra. A similar analysis as done for acridine allows us to determine that the adiabatic electron affinity of phenazine is 1.27 ± 0.01 eV and the S_0_–T_1_ gap is 1.97 ± 0.02 eV. The spectrum at *h*ν = 1.5 eV also allows us to identify vibrational progressions with spacing of 56 ± 2 meV (450 ± 20 cm^–1^) and 81 ± 2 meV (650 ± 20 cm^–1^) for the ground electronic state of the neutral. The inset of the photoelectron spectrum at *h*ν = 1.5 eV again includes a calculated photoelectron spectrum that is in excellent agreement with experiment. The two dominant Franck–Condon active modes can be identified as the υ_7_(a_g_) and υ_15_(a_g_) modes with computed frequencies of 419 and 629 cm^–1^. The displacement vectors of the υ_7_(a_g_) and υ_15_(a_g_) modes are shown in Figure 3b,c, respectively.

The 2D photoelectron spectrum (Figure 1c) again shows clear signs of electronic resonances, with a photoelectron feature at eKE = 0.27 eV (between *h*ν = 1.7 and 3.0 eV) and the spectrum at *h*ν = 2.1 eV in Figure 2b being a representative example. There are also clear changes in the spectra around *h*ν = 2.9 and 3.4 eV. These changes are additionally associated with changes in the angular distributions. Again, the location of resonances is highlighted in Figure 1c and its corresponding 2D β_2_ spectrum in Figure 1f by horizontal dashed lines.

The resonance energies for anthracene, acridine, and phenazine are collected in Table I, where we present the energies in terms of *h*ν as well as in terms of their energy above the neutral state (i.e., *h*ν location minus the electron affinity of respective molecules, *E*_exp_), which corresponds to the resonance energies as viewed from an electron scattering perspective. Table I also includes the resonance energies determined for anthracene from a previous electron transmission spectroscopic study (in brackets),^41^ as well as our calculated results for all three molecules.

**Table I:** Energies of the Resonances Obtained from Experiment (*E*_exp_) and Calculations with the Molecule in Neutral Geometry (*E*_calc,n_) and Anion Geometry (*E*_calc,a_)^a^

**anthracene, C_14_H_10_**						
***D*_*n*_**	***h*ν**	***E*_exp_**	***E*_calc,n_**	***E*_calc,a_**	***E*_calc,sc,a_**	**sym**
π_1_*		–0.53	–0.11	–0.33	–0.61	b_1u_
π_2_*	1.1	0.6 (0.60)	1.07	1.04	0.66	a_u_
π_3_*	1.6	1.1 (1.13)	1.72	1.65	1.23	b_3g_
2p1h		(1.63)				
		(2.15)				
π_4_*	3.0	2.5 (2.63)	2.86	2.86	2.35	b_1u_
π_5_*	3.4	2.9 (2.80)	3.60	3.40	2.85	b_2g_

**acridine, C**_**13**_**H**_**9**_**N**						
***D*_*n*_**	***h***ν****	***E*_exp_**	***E*_calc,n_**	***E*_calc,a_**	***E*_calc,sc,a_**	**sym**
π_1_*		–0.86	–0.45	–0.69	–0.94	b_1_
π_2_*	1.4	0.5	0.96	0.94	0.57	a_2_
π_3_*	1.9	1.0	1.65	1.59	1.17	a_2_
π_4_*	2.6	1.7	2.10	2.10	1.64	b_1_
π_5_*	3.3	2.4	3.26	3.02	2.49	b_1_

**phenazine, C**_**12**_**H**_**8**_**N**_**2**_						
***D*_*n*_**	***h***ν****	***E*_exp_**	***E*_calc,n_**	***E*_calc,a_**	***E*_calc,sc,a_**	**sym**
π_1_*		–1.27	–0.86	–1.10	–1.32	b_1u_
π_2_*	1.6	0.3	0.81	0.81	0.45	a_u_
π_3_*	2.3	1.0	1.56	1.50	1.09	b_3g_
π_4_*	2.9	1.6	1.89	1.84	1.40	b_1u_
π_5_*	3.4	2.1	3.02	2.86	2.35	b_2g_

a *E*_calc,sc,a_ is the scaled energy of the resonance in the anion geometry (see text) and the average difference Δ*E* = *E*_exp_ – *E*_calc,sc,a_ is less than 0.1 eV. All energies are given in eV.

### Computed Resonances of Anthracene, Acridine, and Phenazine Radical Anions

The results from the EOM-CCSD calculations are summarized together with the experimental results in Table I. Results are reported for both the geometries of the ground state of the neutral molecules and the geometries of the ground states of the anions. The ground electronic states of the radical anions of anthracene (X^2^B_1u_), acridine (X^2^B_1_), and phenazine (X^2^B_1u_) are calculated to lie 0.33, 0.69, and 1.10 eV below their respective neutral ground states at the calculated geometries of the anions. These values compare relatively well with the measured electron affinities (0.53, 0.86, and 1.27 eV, respectively). Note that the symmetry labels are defined with the PAH/PANH lying in the *xy* plane with the long axis in the *y* direction.

**Figure 4:**
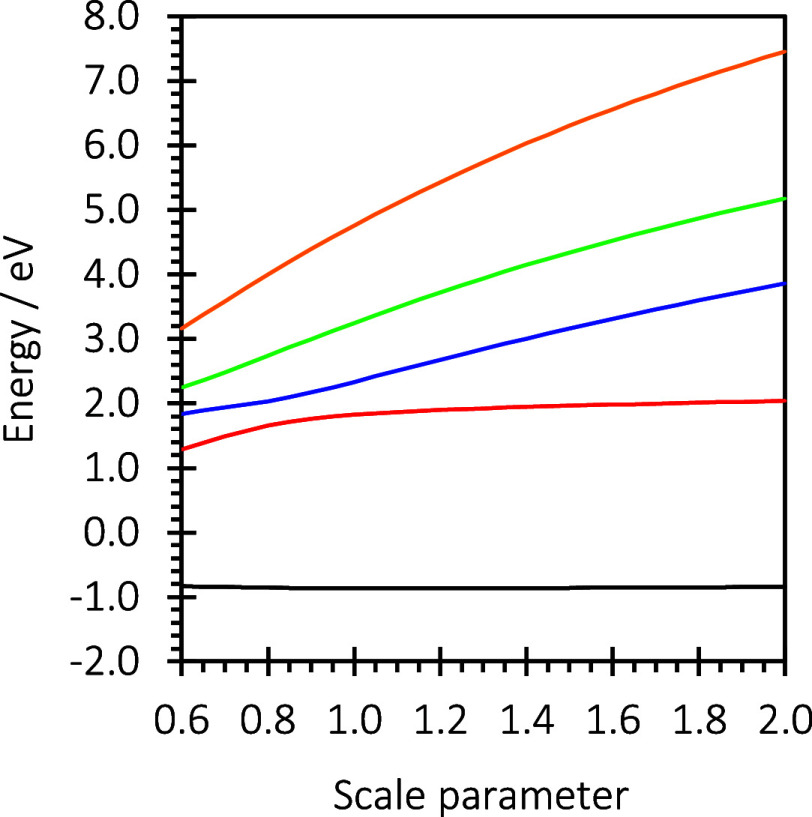
Stabilization graph for the ^2^B_1u_ anion states of phenazine using the geometry of the neutral molecule.

For the unbound anions (i.e., those that lie energetically above the ground state of the neutral molecule), the EOM-CCSD approach was combined with the stabilization method, with exponent scaling and analytic continuation to determine the complex resonance energies. Figure 4 shows the stabilization graph for the ^2^B_1u_ anion states of phenazine with the geometry of the neutral molecule. In addition to the bound state at −0.9 eV, it displays an avoided crossing near 1.9 eV due to a resonance state. Using data from the avoided crossing between the second and third roots (red and blue curves) near a scale parameter of 0.8 gives a resonance energy of 1.89–0.26*i* eV, where the imaginary part corresponds to the half-width.

The energies of the calculated resonance states at the geometries of the anion, *E*_calc,a_, and the geometries of the neutral species, *E*_calc,n_, are in close agreement for the two sets of geometries. The calculated resonances energies tend to be about 0.5 eV higher than the measured values for anthracene, acridine, and phenazine, respectively. The discrepancies between the calculated and measured experimental resonance positions are expected to be primarily due to limitations of the basis set used, although correlation effects not recovered with the EOM-CCSD method could also be a factor. EOM-CCSD has been applied to a wide range of bound and temporary anions. When used with a flexible basis, EOM-CCSD generally gives electron attachment energies within 0.2–0.3 eV of the experiment, placing the anion states too high in energy (compared to the ground state of the neutral species; see, e.g., Falcetta et al.^37^). The discrepancies in the calculated electron attachment energies are somewhat larger in the present case due to the limitations of the basis set used. We note that a scaled energy, *E*_calc,sc,a_, using the expression
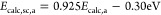
gives anion energies within 0.1 eV of the experimental photoelectron values on average. These scaled values are also included in Table I. Hence, despite this systematic error, the overall computational results accurately captured the resonances in all three systems.

In the case of anthracene, the electron transmission spectrum^36^ also displays a pronounced feature at 1.6 eV and a very weak shoulder at 2.15 eV that appear not to be due to 1p resonances. The 1.6 eV resonance has also been observed in vibrational excitation spectra^20^ and has been assigned to the lowest energy 2p1h resonance, which is dominated by the HOMO^–1^(LUMO)^2^ configuration.^41^ There is also evidence for the 2p1h resonance in the absorption spectrum in a cold molecular matrix; the small peak at 2.1 eV aligns well with the excitation energy expected for the 2p1h state from the anion. The EOM-CCSD procedure starting from the ground state of the neutral molecule cannot accurately characterize 2p1h anion states. To circumvent this problem, we calculated the energy of the ^2^B_1u_ ground state anion and the lowest energy ^2^B_2g_ 2p1h excited state anion using the ionization potential EOM-CCSD method starting with the (LUMO)^2^ dianion, employing the cc-pVDZ(-f,-d) basis set, which avoids the diffuse functions that would cause collapse onto a DC level. These calculations place the ^2^B_2g_ 2p1h anion only 2.7 eV above the ground state anion and only a few tenths of an eV above the T_1_ state of the neutral molecule. In addition, we find that there is a large ∼0.6 eV energy lowering upon geometry relaxation of the anion in the 2p1h state. We also calculated harmonic vibrational frequencies of the ^2^B_2g_ 2p1h anion and the T_1_ state using the B3LYP density functional method. Combining the results of these calculations, we conclude that the zero-point level of the ^2^B_2g_ 2p1h anion state lies below the zero-point level of the T_1_ state of the neutral.

With regard to whether the present experiment provides evidence for the lowest energy 2p1h anion state of anthracene, we note that there is a slight change in the relative photoelectron signals (Figure 1a) around *h*ν = 2.1 eV, which is where we would expect the 2p1h resonance to lie (i.e., *E*_exp_ = *h*ν – EA = 2.1–0.53 = 1.6 eV). However, there is no clear signature of a noticeable change in associated dynamics and therefore we did not extract a resonance in our previous work at this energy.^28^ The lack of clear signature highlights a limitation of 2D photoelectron spectroscopy when used in isolation; it would be beneficial to additionally measure the photodetachment action spectrum across the energy range, as recently done for tetracene anions for example.^61^

As noted above, the electron transmission spectrum of anthracene also reveals a very weak shoulder at 2.15 eV. While this was assigned to a 2p1h resonance state in ref (41), this feature is not observed in the vibrational excitation spectrum from Allan.^20^ Hence, we question whether it actually corresponds to a separate resonance state.

### Effect of N Atom Substitution

**Figure 5:**
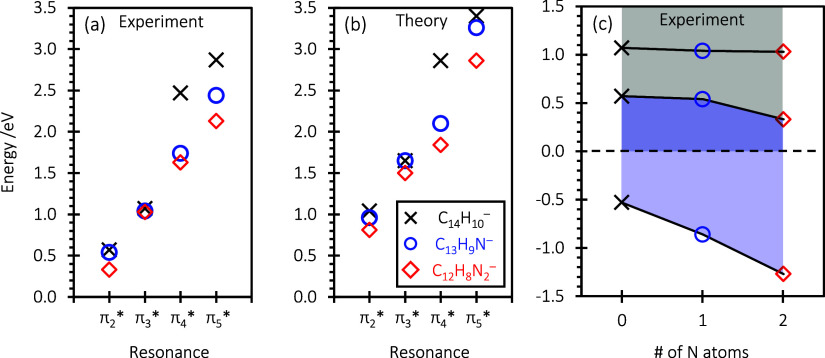
Energies of the resonances obtained from experiment (a) and calculations (b); energies of the ground state and lowest two resonances as a function of N atoms in the PAH, taken from the experiment (c).

We now turn to the comparison between the PAH/PANHs and consider the effect of substituting N atoms on the energetics, both experimentally and computationally. First, the experiments establish that the electron affinity (associated with formation of the ground state anion) increases with the substitution of N atoms. When going from anthracene to acridine, the electron affinity increases by 0.33 eV and to phenazine by 0.74 eV. In contrast, the electronic resonances do not appear to be particularly sensitive to the substitution, especially for the first two resonances. The first resonance, π_2_*, decreases in energy from 0.6 to 0.3 eV (calculated from 1.04 to 0.81 eV) with the substitution of the two N atoms. Similarly, the second resonance, π_3_*, decreases from 1.1 to 1.0 eV (calculated 1.65 to 1.50 eV). We collate the location of the resonances into energy level diagrams in Figure 5 using the experimental (a) and computational (b) data. This clearly shows the small variations between the resonances for the different species and underscores that the calculations captured the overall trends.

Figure 5 also shows that for the higher-lying resonances, there are clear differences in the resonance energies of the three molecules. Specifically, the energy for the resonance associated with π_4_*, in anthracene is 0.7 eV higher in energy than acridine and 0.8 eV compared to phenazine. For this resonance, the addition of one N atom has a significant stabilizing effect but the addition of a second much less so. The final resonance we consider, π_5_*, again shows an effect of substituting one N atom, but less so than for the π_4_* resonance, and the addition of a second N atom has a similar stabilizing effect as the first.

Note that one would also expect the lowest energy 2p1h anion states of acridine and phenazine to fall near 1.6 eV. However, in these species, a 2p1h anion at this energy would be overlapping a 1p anion state, and therefore, we do not consider the 2p1h anion states of these species here.

## Discussion

**Figure 6:**
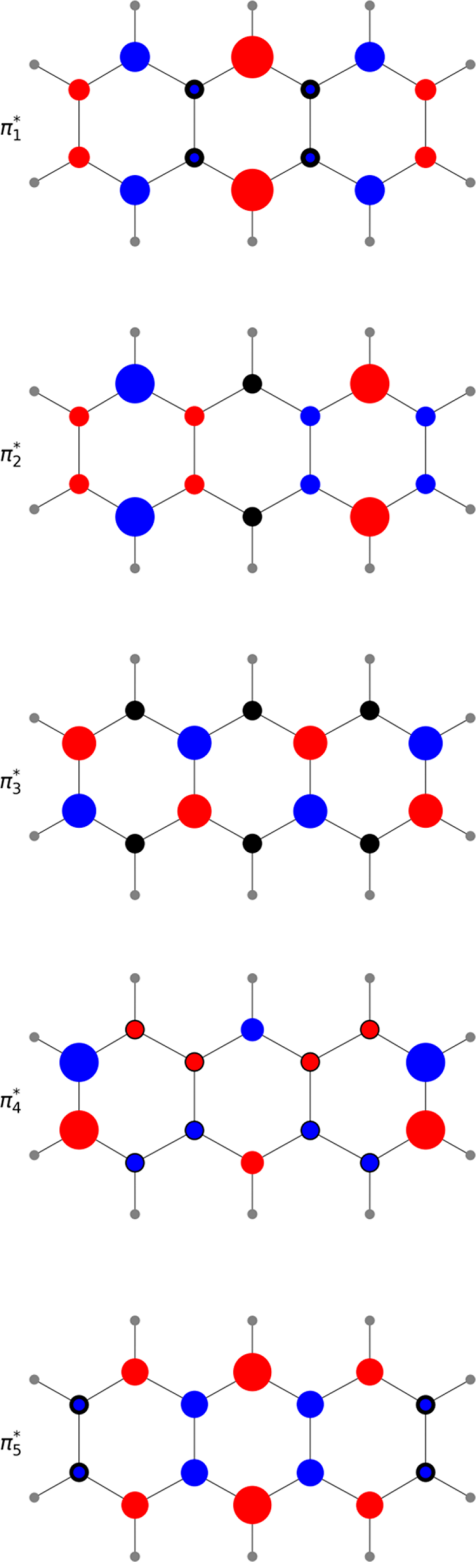
Five lowest unoccupied molecular orbitals calculated using Hückel theory. Addition of an electron to the π_1_* orbital leads to the anion’s ground state. The addition to an electron in the π_2_* or π_3_* orbitals leads to the two lowest-lying resonances. Within the context of Hückel theory, substitution of the central C atoms with N atoms decreases the energy of the π_1_*, π_4_*, and π_5_* orbitals but not the π_2_* or π_3_* orbitals as these have a node through the central atoms.

The effects of N atom substitution on the anion energies of acridine and phenazine can, to a large extent, be explained by using a simple Hückel model. The lowest five unoccupied molecular orbitals calculated using Hückel theory for anthracene are shown in Figure 6. The π_1_* molecular orbital of anthracene (LUMO) has sizable electron density residing on the central atoms, and consequently, the π_1_* orbital energy decreases with N atom substitution of these atoms. Hence, the overall trend of increasing electron affinity is consistent with decreasing π_1_* orbital energy.

The π_2_* and π_3_* molecular orbitals are degenerate for anthracene within Hückel theory. This is an accidental degeneracy, due to the approximations inherent in Hückel theory, for example, the assumption that all C atoms have the same α value, and in the actual molecule, the associated anion states (^2^A_u_ and ^2^B_3g_, respectively) are nondegenerate. Importantly, both the π_2_* and π_3_* orbitals have a node that passes through the central C atoms (Figure 6). Exchanging these C atoms with one or two N atoms, therefore, does not lead to an appreciable shift in the energies of these anion states. Indeed, this is what is observed experimentally and seen from our detailed calculations (Figure 5 and Table I). Specifically, double N atom substitution stabilizes the π_2_* and π_3_* resonances by only ∼0.3 eV compared to those of anthracene. That is to say, the electron impact resonances associated with π_2_* and π_3_* will be expected to be found at similar energies for anthracene, acridine, and phenazine. Note that, due to N-atom substitution, the excitation energy from the anion ground state has increased significantly, but this is predominantly driven by the increase in electron affinity of the ground state. This is contrary to what is observed when considering the effect of solvation, where the various π* anion states are stabilized to approximately the same extent.^46,62,63^

The next two molecular orbitals of anthracene (π_4_* and π_5_*) in the Hückel model are also degenerate. However, these orbitals have electron density residing on the central C atoms (Figure 6). Hence, one might intuitively expect that N atom substitution here will affect the resonance energies (as is the case for the π_1_* orbital and thus the ground state). This expectation is in agreement with observations where the energy of the π_4_* resonance is stabilized by 0.9 eV from anthracene to phenazine and 0.8 eV for the π_5_* resonance and the overall affect can be clearly seen in Figure 5a,b. The lowest energy 2p1h anion state would also be expected to shift in energy due to N atom substitution since the HOMO has nonzero coefficients on the N atoms.

The stabilization of the π_4_* and π_5_* resonances in the N-substituted PAH compared to those in anthracene suggests that the density of resonances will be higher in these PANHs than in anthracene. From an electron capture perspective to form the corresponding ground state radical anion, this increase in resonance density is expected to lead to an increased electron capture probability.^17,59^ However, the fact that the π_2_* and π_3_* resonances are not stabilized to a significant extent may counterbalance this expectation. Specifically, while the π_2_* and π_3_* resonances remain at the same (approximate) energy above the neutral, the anion ground states are decreasing in energy (increasing EA), thus leading to an overall increase in the energy gap between the lowest energy resonance and the ground state. For anthracene, the gap between the ^2^A_u_ and X^2^B_1u_ anion states is 1.1 eV, increasing to 1.6 eV for phenazine. Within a simple energy gap law, the internal conversion rate may therefore be expected to be reduced in phenazine, and assuming a constant autodetachment rate, this would lead to a lower yield of ground state anion formation.

Figure 1 is broadly consistent with the above considerations. First, we observe that for all three systems, population appears to become trapped in the π_2_* resonance with autodetachment producing electrons with constant kinetic energy (i.e., internal energy is conserved in the autodetachment) at eKE ∼ 0.5, 0.4, and 0.3 eV for anthracene, acridine, and phenazine, respectively. This bottleneck is observed for an energy range spanning over 1 eV from the onset of the π_2_* resonance. Even though anthracene has the highest-lying π_2_* resonance of the three systems, it is the only one that shows clear evidence of ground state formation (through thermionic emission), albeit arising from the π_3_* resonance.^28^ This is broadly consistent with the fact that anthracene has the smallest energy gap to the ground state and, within an energy gap law, the largest rate of ground state formation.

The simple energy gap law, however, does not account for the dynamics that take place on potential energy surfaces and the possibility of conical intersections that funnel population transfer, and therefore, it is important to appreciate the limitations of such an interpretation. Indeed, phenazine also appears to show additional dynamics across the π_2_* and π_3_* resonances with some electron signal appearing at even lower eKE than the signal arising from the π_2_* resonance bottleneck. This signal also extends to eKE = 0 eV. However, the appearance of the very low-energy signal in phenazine (shown in Figure 2b at *h*ν = 2.1 eV) is different from that of anthracene which shows the expected exponentially decaying (Boltzmann) photoelectron distribution associated with thermionic emission.^18,64,65^ This difference might reflect dynamics taking place in either the π_2_* and π_3_* resonances, where autodetachment along some coordinate leads to a broad distribution of outgoing electron eKE. We also note that the π_2_* resonance is lower in energy for phenazine than it is for either anthracene or acridine (see Figure 5c) and this might affect the autodetachment lifetime. Specifically, the centrifugal barrier to emission (expected to be *l* = 3) will inhibit autodetachment more at lower electron energies, such that we might expect the autodetachment rate for phenazine to be lower (i.e., slower autodetachment) than the other two anions.

## Conclusions

The electron impact resonances of anthracene, acridine, and phenazine have been studied using 2D photoelectron imaging of their corresponding anions. The 2D photoelectron spectra show clear evidence of a number of resonances and these have been assigned with the aid of ab initio calculations. For anthracene, an updated assignment of some resonances that have previously been observed in electron transmission and vibrational excitation spectra is offered. In particular, a 2p1h resonance at ∼1.6 eV that was previously assigned is verified in the current calculations although its presence is not immediately visible in the 2D photoelectron spectrum. The very weak feature seen in electron transmission spectra around 2.1 eV, which is not observed in vibrational excitation, is not considered to be an anion resonance.

The effect of substituting the central C atoms of anthracene with N atoms on the temporary anion resonances was considered. Due to the presence of the nodes in the π_2_* and π_3_* molecular orbitals at the substitution sites, the energies of lowest two resonances do not change appreciably with the substitution of the N atoms. The electron affinity associated with the ground state anion, on the other hand, increases significantly upon the N atom substitutions. The net effect is that the energy gap between the lowest two resonances and the (bound) ground electronic state of the anion increases with the substitution of N atoms. Based on a simple energy gap law argument, the internal conversion rate to form the anion ground state therefore decreases with an increasing number of N atoms, making electron capture less likely, which is broadly consistent with our observations. This conclusion contrasts with the effect of solvation on resonances, for which the various anion states are stabilized to a similar extent.^46^ The conclusion that an increasing electron affinity does not necessarily lead to an increased electron capture probability is interesting because, in models that consider the charge balance in interstellar dense molecular clouds, this assumption is often made.^4−7^ Knowledge of how the energies of various anion states shift upon chemical substitution is relevant to the design of molecular electronics materials.
